# VprBP (DCAF1): a promiscuous substrate recognition subunit that incorporates into both RING-family CRL4 and HECT-family EDD/UBR5 E3 ubiquitin ligases

**DOI:** 10.1186/1471-2199-14-22

**Published:** 2013-09-13

**Authors:** Tadashi Nakagawa, Koushik Mondal, Patrick C Swanson

**Affiliations:** 1 Department of Cell Proliferation, United Center for Advanced Research and Translational Medicine, Graduate School of Medicine, Tohoku University, Sendai 900-8575, Japan; 2 Department of Medical Microbiology and Immunology, Creighton University, 2500 California Plaza, Omaha, NE 68178, USA

**Keywords:** VprBP, DCAF1, DDB1, Cul4, CRL4, EDD, UBR5, Dyrk2, Merlin, Katanin, UNG2, LGL2, Mcm10, Histone H3, RORα, Methyl degron, p53, TERT, telomerase, RAG1, V(D)J recombination, HIV, Vpr, Vpx, UL35, Ubiquitin, E3 ubiquitin ligase, RING, HECT, WD40 repeat

## Abstract

The terminal step in the ubiquitin modification system relies on an E3 ubiquitin ligase to facilitate transfer of ubiquitin to a protein substrate. The substrate recognition and ubiquitin transfer activities of the E3 ligase may be mediated by a single polypeptide or may rely on separate subunits. The latter organization is particularly prevalent among members of largest class of E3 ligases, the RING family, although examples of this type of arrangement have also been reported among members of the smaller HECT family of E3 ligases. This review describes recent discoveries that reveal the surprising and distinctive ability of VprBP (DCAF1) to serve as a substrate recognition subunit for a member of both major classes of E3 ligase, the RING-type CRL4 ligase and the HECT-type EDD/UBR5 ligase. The cellular processes normally regulated by VprBP-associated E3 ligases, and their targeting and subversion by viral accessory proteins are also discussed. Taken together, these studies provide important insights and raise interesting new questions regarding the mechanisms that regulate or subvert VprBP function in the context of both the CRL4 and EDD/UBR5 E3 ligases.

## Introduction

Virtually all cellular processes are subjected to some level of regulation by the ubiquitin modification system which mediates the attachment of ubiquitin or ubiquitin-like molecules to proteins in the involved pathways (for review, see [1]). It is also now appreciated that this system can be subverted by pathogens to disable host responses and alter the cellular environment to benefit the microorganism (for reviews, see [2-4]). Ubiquitin is a 76 amino acid protein that is covalently attached to a target protein through a series of enzymatic steps in which free ubiquitin is initially coupled to an activating enzyme (E1) in an ATP-dependent reaction, and then transferred to the catalytic cysteine of a conjugating enzyme (E2). In the last step, the ubiquitin-linked E2 associates with an ubiquitin ligase (E3), which catalyzes the transfer of ubiquitin to an ϵ-amino group of a lysine residue in the targeted protein. Additional ubiquitin molecules may be appended to lysine residues in ubiquitin to form polyubiquitin chains. The number of attached ubiquitin molecules and the specific lysine residue(s) used to link them together dictate whether the outcome of ubiquitination mainly serves to alter the function of the target protein, or triggers its degradation through the proteasome pathway.

There are two major classes of E3 ubiquitin ligases (termed E3 ligase henceforth) that differ in how they mediate ubiquitin transfer (for reviews, see [1,5]). Those that contain a RING (*r*eally *i*nteresting *n*ew *g*ene) domain, or the related U-box domain, facilitate the direct transfer of ubiquitin from the ubiquitin-E2 conjugate to the target protein without forming a covalent intermediate. By contrast, those with a HECT (*h*omologous to *E*6-AP *c*arboxy *t*erminus) domain first undergo a trans-thioesterification reaction which transfers ubiquitin from the E2 enzyme to an active site cysteine residue in the HECT domain before the ubiquitin is ultimately coupled to the target protein. Most eukaryotic organisms have only a single E1 activating enzyme, but express tens of E2 conjugating enzymes and several hundred or more E3 ligases. Substrate specificity is largely determined by the E3 ligase; however, the substrate binding and catalytic activity of a given E3 ligase may or may not be found within the same molecule. The cullin RING ligases (CRLs) are a large group of E3 ligases with separable substrate binding and catalytic activities (for review, see [6,7]). These E3 ligases have a modular organization in which one of the cullin family members of scaffold proteins binds both a small RING-containing catalytic subunit (Roc1 [*r*egulator *o*f *c*ullins 1] or Roc2; also called Rbx1 [*R*ING *b*o*x* protein] or Rbx2), and a cullin-specific adaptor protein. In most cases, the adaptor protein, in turn, binds a substrate recognition subunit that recruits and positions the substrate in proximity to the catalytic subunit for ubiquitination. The CRL is defined by the cullin scaffold protein, of which there are seven members in humans and mice (i.e. CRL1 contains Cul1). Because the CRL adaptor protein is generally cullin-specific, it is often not included in the designation, but the substrate recognition subunit is included after the CRL in superscript. For example, Damaged DNA *b*inding protein 1 (DDB1) is the adaptor protein for the CRL4 E3 ligase. The *D*DB1-*C*ul4 *a*ssociating *f*actors (DCAFs) that comprise the substrate recognition subunits for the CRL4 E3 ligase are indicated as CRL4^DCAF^. This convention will be followed here.

The HECT-domain E3 ligases are characterized by the presence of a C-terminal HECT domain (for review, see [8]). This family has been further divided into three broad subgroups based on the presence or absence of additional WW or RCC1 (*r*egulator of *c*hromatin *c*ondensation 1)-like domains (RLDs) in the amino-terminal region of the protein. These include the Nedd4/Nedd4-like subgroup which contains WW domains (identified by a signature pair of tryptophan residues), the HERC (*HE*CT and *RC*C1-like domain) subgroup which contain RLDs, and a subgroup which harbors neither WW domains nor RLDs (non-WW/non-RLD). In contrast to the CRL family of E3 ligases, HECT-domain E3 ligases more commonly function as a single subunit E3 ligase, mediating both substrate recognition and ubiquitination. However, for some HECT E3 ligases, such as Nedd4, adaptor proteins may be engaged to mediate substrate recruitment or influence the subcellular distribution of the E3 ligase to direct ubiquitination of localized substrates [9]. The degree of flexibility of adaptor proteins and substrate recognition subunits to service multiple E3 ligases is important for understanding how the substrates they recruit are regulated in a variety of spatial and temporal contexts.

**Figure 1:**
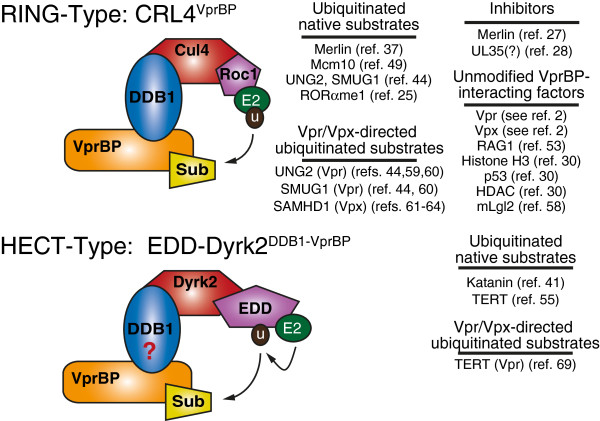
**VprBP services two distinct E3 ubiquitin ligases.** Some VprBP-interacting proteins are normally subjected to VprBP-dependent ubiquitination in unperturbed cells (native), whereas others are native or novel substrates that undergo accelerated Vpr- or Vpx-dependent degradation in the context of CRL4^VprBP^ or EDD-Dyrk2^DDB1-VprBP^ complexes. Merlin and UL35 may act to inhibit the CRL4 ligase. For several VprBP-interacting proteins, no evidence of ubiquitin modification has been reported. In some of these examples (e.g. mLgl2), the identity of the VprBP-associated E3 ligase has not been formally established. DDB1 may or may not physically link the EDD-Dyrk2 E3 ligase to substrates through VprBP in all cases. For additional details, see text.

Here we review and discuss the discovery and characterization of *V*iral *p*rotein *R b*inding *p*rotein (VprBP, also called DCAF1), and its emerging role as a dual-purpose substrate recognition subunit for two distinct E3 ligases: the RING-family member CRL4 and the “non-WW/non-RLD” HECT-family member EDD/UBR5 (*E*3 ligase identified by *d*ifferential *d*isplay/*ub*iquitin protein ligase E3 component n-*r*ecognin 5) (Figure 1). Regulatory roles of the CRL4 and EDD/UBR5 E3 ligases for which there is no known involvement of VprBP as a substrate recognition molecule will not be discussed in depth here.

### VprBP: discovery and early association with the CRL4 E3 ligase

In addition to the main Gag, Pol, and Env open reading frames (ORFs), primate lentiviruses, including human immunodeficiency virus 1 (HIV-1), have additional ORFs encoding a variety of other regulatory and accessory factors, one of which is named Viral protein *R* (Vpr). By the early 1990s, Vpr was known to play a key role in promoting viral replication but its function remained enigmatic. Hypothesizing that Vpr targets a host protein to mediate its function, Zhao *et al.* used purified, bacterially expressed Vpr as bait to isolate Vpr-interacting proteins from HeLa cell nuclear extracts by co-immunoprecipitation (co-IP) [10]. This screen yielded a protein initially called Vp*r i*nteracting *p*rotein (RIP), which was renamed VprBP in their subsequent study in which the authors cloned the VprBP cDNA, mapped the Vpr-interacting region to residues 636–1507 of VprBP [11], and showed that VprBP binding to Vpr promotes cytoplasmic retention of Vpr. However, the normal function of VprBP and the significance of VprBP-mediated cytoplasmic sequestration of Vpr for HIV replication remained unclear.

A major breakthrough in understanding the normal role of VprBP came from proteomic screens designed to identify DDB1-associated substrate recognition subunits for the CRL4 E3 ligase [12-15]. VprBP was identified in three of these studies [12-14] (called DCAF1 by Jin *et al*. [12] and Angers *et al*. [13]). Most identified DDB1-interacting proteins share a common structural motif called a WD40 domain (for reviews, see [16,17]). The WD40 domain contains multiple WD40 repeats (ranging from 4–16), each spanning 40–60 residues and bearing a signature Trp-Asp (WD) dipeptide at its C-terminus, although the Trp-Asp dipeptide is sometimes substituted by Tyr-Asp, Ile-Asp, or Trp-Cys in the DCAFs. In most DCAFs, including VprBP, the Trp-Asp dipeptide is also followed by an X-Arg (or occasionally X-Lys) dipeptide. This sequence, designated a WDXR motif, is often evolutionarily conserved between orthologous DCAFs, and mutational analysis suggests that the basic residue within the WDXR motif is required for stable association of DCAF proteins to DDB1. Each WD40 repeat is composed of four anti-parallel beta-strands that form a beta-propeller; consecutive beta-propellers are connected by a peptide linker.

DDB1 itself contains three WD40 beta-propeller domains (BPA, BPB, and BPC) and a C-terminal helical domain [13]. Crystal structures of DDB1 bound to different substrate recognition subunits, including DDB2 [18-20] and Cockayne Syndrome A (CSA) [18], reveal a common mode of binding in which the WD40 beta-propeller domain in DDB2 and CSA stabilizes association with DDB1 by mediating hydrophobic contacts with the BPA domain of DDB1 and by anchoring an amino-terminal helix-loop-helix motif that is inserted into a cavity at the interface between the BPA and BPC domains of DDB1. Notably, some viral proteins, such as hepatitis B virus X protein, are able to subvert the CRL4 E3 ligase by using an alpha-helical motif to bind DDB1 at the BPA-BPC interface in a manner analogous to cellular CRL4 substrate receptors [21]. Whether VprBP shows a mode of DDB1 binding similar to DDB2 and CSA remains unclear, as no readily identifiable counterpart to the helix-loop-helix motif in DDB2 and CSA has been detected in VprBP [18,21]. VprBP preferentially binds DDB1 associated with a form of Cul4 that has been post-translationally modified, most likely by NEDD8 although this was not experimentally confirmed [22]. In the next sections, we will review current knowledge regarding VprBP domain structure and organization, discuss the different cellular processes regulated by VprBP through its association with the CLR4 E3 ligase and its more recently uncovered affiliation with the EDD/UBR5 E3 ligase, and highlight recent insights on the effects of VprBP targeting by viral accessory proteins.

### VprBP: domain organization and structural features of VprBP-DDB1 and CRL4^VprBP^ complexes

**Figure 2:**
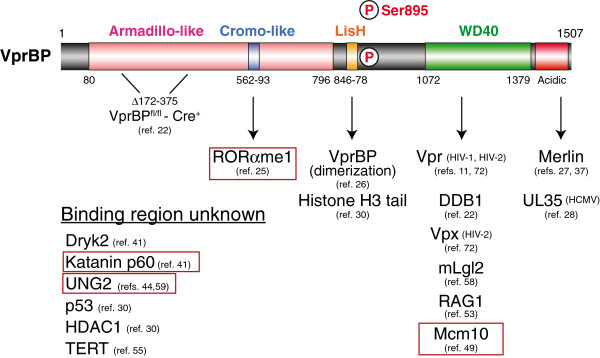
**VprBP structural motifs and interacting proteins.** The 1507 amino acid human VprBP isoform encoded by transcript variant ENST00000563997 in the Ensembl database (release 71) [29] is shown with the domain features for this isoform as described in the database or in the text. VprBP-interacting proteins are identified below the diagram; those for which the binding site has been mapped are shown below the region mediating the association. Proteins targeted for VprBP-dependent ubiquitination are boxed. The region removed after Cre-mediated deletion of a conditional allele [22], and the location of a DNA-PK-dependent phosphorylation site [30] are also indicated.

The human and murine VprBP genes express two or three predicted spliced transcript variants encoding distinct isoforms. The most similar and best characterized human and murine isoforms of VprBP are 1507 and 1506 amino acids, respectively, and share 98% identity. A predicted Armadillo-type fold domain encompasses most of the amino-terminal half of the protein (residues 80–796) (see Figure 2). This region consists of tandemly arrayed Armadillo repeat motifs, each about 40 amino acids in length, which are predicted to form layers of alpha helices that assemble into a right-handed superhelix [23,24]. These structures present a large solvent-accessible surface area well-suited for mediating contact with interacting proteins. Within this region lies a small chromo-like domain (residues 562–593), similar to those found in chromatin remodeling proteins, that has recently been implicated in mediating interactions with monomethylated proteins ( [25], see below). A central region, designated by its homology to Lis1 (LisH; residues 846–878), contains a L-*X*_2_-L-X_3-5_-L-X_3-5_-L sequence motif that adopts an alpha-helical conformation and is required to mediate oligomerization of VprBP [26]. The carboxyl-terminal half of the protein contains a WD40 domain consisting of four WD40 repeat motifs, which, as discussed above, not only mediate interactions with DDB1, but also support contacts with other substrate and interacting proteins (see below). This region is followed by a stretch of ~100 amino acids rich in acidic residues that may interact with factors that disrupt or subvert the activity of the associated E3 ligase [27,28]. Notably, the acidic region is absent from a shorter murine isoform of VprBP.

To gain insight into the structural and conformational features of VprBP-containing DDB1 and CRL4 complexes, Ahn *et al.* used *s*ize-*e*xclusion chromatography coupled to in-line *m*ulti-*a*ngle *l*ight *s*cattering (SEC-MALS) and SDS-PAGE to analyze fully assembled and purified DDB1-VprBP and CRL4^VprBP^ complexes prepared with a form of VprBP lacking the Arm-like domain (VprBP_817-1507_) [26]. The authors elegantly showed that both complexes contained equimolar amounts of each of the individual proteins, and had an apparent mass that was approximately twice that predicted based on the molecular weight of each subunit, leading them to conclude that both complexes assemble dimeric structures. Further examination of CRL4^VprBP^ complexes by electron microscopy revealed evidence of two-fold rotational symmetry in the structures. A comparison of the *in vitro* ubiquitination activity of CRL4^VprBP(817–1507)^ and CRL4^VprBP(1005–1507)^ (which lacks the LisH motif) showed that CRL4^VprBP(817–1507)^ was at least 2-fold more efficient, suggesting that VprBP-mediated dimerization promotes CRL4^VprBP^ ubiquitin transfer activity.

VprBP may also be subjected to post-translational modification. Kim *et al*. reported that DNA-PK phosphorylates VprBP *in vitro* at Ser895, and showed that phospho-Ser895-specific polyclonal antibodies detect phosphorylated VprBP in U2OS cells after etoposide-induced DNA damage [30]. As discussed below, phosphorylation at this site is reported to alleviate VprBP-mediated repression of p53-dependent transcription.

### Physiological roles and binding partners of VprBP

VprBP has been implicated in regulating a variety of normal cellular processes, including proliferation, DNA replication, cell cycle progression, telomere maintenance, DNA damage responses, and competition between neighboring cells. The evidence supporting a role for VprBP in these processes, and the context and targets of the associated E3 ligase machinery, where known, are discussed in the following sections.

#### Proliferation, DNA replication, and cell cycle

Proliferating cells undergo repeated cycles of DNA replication (DNA synthesis or S phase) and cell division (mitosis or M phase), which are temporally separated by gap phases (G1 or G2 phases) (for review, see [31]). Potential replication initiation sites, called replication origins, are marked, or “licensed”, by the formation of a pre-replication complex (pre-RC) that includes the ORC1-6, CDT1, CDC6, and MCM2-7, beginning late in M phase and proceeding through the G1 phase. During S phase, pre-RCs (30,000-50,000 in mammals) are then activated, or “fired”, following recruitment of DNA replication machinery such as DNA polymerases. DNA replication propagates bidirectionally from the origins until the whole genome is precisely duplicated. Importantly, not all origins are used at the very onset of S phase, but origins are fired in a temporal order by which DNA replication is regulated during S phase [32,33]. Precise DNA replication is of utmost importance to transmit genetic information intact to daughter cells. High fidelity DNA duplication is ensured by S phase checkpoint activation, which inhibits late origin firing in a transient manner to provide time for DNA repair when cells encounter DNA damage during S phase. If damaged DNA is not repaired, cells exit S phase and undergo G2 arrest [34,35].

Recent studies by McCall *et al.* provide several lines of evidence suggesting VprBP is involved in regulating DNA replication [22]. First, silencing VprBP expression was shown to suppress proliferation in U2OS and Rb-inactivated (E7 transduced) WI38 cells and increase the percentage of cells in the S and G2 phases in HeLa cells. Second, VprBP and Cul4A were found to exhibit cell cycle-dependent binding to chromatin in HeLa cells, with the association being primarily restricted to the early S and G2 phases. Third, also in HeLa cells, BrdU labeling studies showed that VprBP silencing markedly reduced DNA replication in middle to late S phase. These cells were not responsive to S-phase inhibitors, indicating that DNA synthesis was completely blocked after VprBP silencing. Fourth, DNA fiber-labeling experiments provided evidence that VprBP silencing in HeLa cells increases the frequency of firing of new replication origins, but does not change the frequency of replication termination or dramatically alter the rate of DNA synthesis. To reconcile the seemingly opposing effects of VprBP silencing on BrdU incorporation during S phase and replication origin firing and DNA synthesis rates, McCall *et al*. suggested that VprBP functions to either stabilize the replication fork or regulates the temporal order of early and late origin firing during S phase [22]. The replication defects observed by BrdU labeling experiments in HeLa cells were further confirmed in primary mouse embryonic fibroblasts (MEFs) in which VprBP expression was conditionally disrupted using a Cre-loxP approach. However, in this case, MEFs lacking VprBP did not accumulate in S phase, but were instead reduced in frequency. This outcome was attributed to an increase in apoptosis observed in VprBP-deficient MEFs as assessed by Annexin V staining. The precise mechanism for how VprBP facilitates DNA replication and S phase transit in cells remains unclear, as neither the E3 ligase nor the putative substrate(s) that VprBP recruits to it for ubiquitination were established in this study.

Merlin (also called schwannomin) is a tumor suppressor encoded by the *n*eurofibromatosis type 2 (NF2) gene. Inactivating NF2 mutations have been identified in variety of nervous system tumors including schwannomas, ependymomas, meningiomas and mesotheliomas [36]. To determine the mechanism by which Merlin exerts its anti-proliferative activity, two different groups employed *t*andem *a*ffinity *p*urification and *m*ass spectrometry (TAP-MS) to identify novel binding partners of Merlin, with both groups identifying DDB1 and VprBP as Merlin-interacting proteins [27,37]. In both studies, pull-down experiments established that Merlin associates with the CRL4^VprBP^ E3 ligase complex, and were used to map Merlin interactions to the far C-terminal acidic region of VprBP (Figure 2). Li *et al.* went further by showing that Merlin specifically co-IP’s with the unique components of the CRL4^VprBP^ E3 ligase, but not the alternative EDD-Dyrk2^DDB1-VprBP^ E3 ligase, from nuclear-soluble, but not cytoplasmic/membrane, cell fractions, leading the authors to conclude Merlin translocates to the nucleus to engage CRL4^VprBP^[27]. Furthermore, this group found that Merlin association with VprBP depended on maintaining Merlin in a “closed” conformation. This closed and active form of Merlin can be converted to an “open”, inactive form by Ser518 phosphorylation, which disrupts an intramolecular interaction between the N- and C-terminal regions of Merlin [38,39].

Using a transient overexpression system, Huang and Chen showed that VprBP downregulates Merlin in a dose-dependent manner by triggering its ubiquitination, and that this effect could be partially overcome by proteasome inhibition [37], leading this group to conclude Merlin is targeted for degradation by CRL4^VprBP^ E3 ligase. However, this conclusion has been challenged by Li *et al.*, who provided evidence that Merlin actually inhibits ubiquitination mediated by the CRL4^VprBP^ E3 ligase [27]. Specifically, levels of VprBP-associated ubiquitination products were shown to diminish as a function of Merlin expression using a Cos7 cell transfection system. Conversely, levels of these products were elevated when Merlin expression was silenced, or the Merlin binding site at the C-terminus of VprBP was removed (VprBP_1-1417_). These authors also showed that silencing Merlin accelerates proliferation in a normal human Schwann cell line or human umbilical vein endothelial cells, but this effect is largely reversed by concomitant VprBP silencing. Finally, genetic evidence for Merlin’s function as a CRL4^VprBP^ inhibitor comes from the author’s characterization of a panel of tumor-derived mutations, which fell into three catagories: those that impair Merlin’s ability to translocate into the nucleus, those that fail to bind VprBP, and those that bind VprBP, but do not inhibit CRL4^VprBP^ activity.

Another cell cycle-related protein regulated by VprBP is the microtubule-severing enzyme katanin, which is composed of a regulatory p80 subunit and a catalytic p60 subunit [40]. The association between VprBP and the catalytic subunit of p60 katanin was discovered by Maddika and Chen through a TAP-MS screen for potential substrates of the *d*ual specificity t*y*rosine-phosphorylation-*r*egulated *k*inase 2 (Dyrk2), which was of interest for its involvement in various signaling pathways activated during cellular, developmental, and oncogenic processes [41]. This screen identified the HECT E3 ligase EDD (UBR5), DDB1, and VprBP as predominant Dyrk2-associated proteins. Co-IP experiments confirmed these interactions and further excluded Cul4 and Roc1 as binding partners, and also established that Dyrk2 kinase activity is dispensable for binding VprBP. Dyrk2 silencing abolished VprBP association with EDD, but not DDB1, suggesting that Dyrk2 functions as an adaptor bridging EDD to the DDB1-VprBP complex. Moreover, Dyrk2 protein levels were not altered by DDB1, VprBP, or EDD silencing, nor did they fluctuate during cell cycle, suggesting that Dyrk2 did not undergo ubiquitin-dependent degradation mediated by EDD. Alternatively, katanin p60 was considered a potential substrate of the EDD-Dyrk2^DDB1-VprBP^ E3 ligase because its homologue in *Caenorhabditis elegans*, MEI-1, was previously shown to undergo phosphorylation-dependent ubiquitin-mediated degradation by the Dyrk2 homologue MBK2 during meiotic maturation in *C. elegans*[42]. In support of this possibility, Maddika and Chen demonstrated that katanin p60 levels in HeLa cells were increased by silencing of EDD, Dyrk2 or VprBP, and were decreased when these molecules were overexpressed [41]. However, overexpressing a kinase-inactive form of Dyrk2 had no effect on katanin p60 levels, suggesting its degradation depends on Dyrk2-mediated phosphorylation. Consistent with these results, the EDD-Dyrk2^DDB1-VprBP^ complex was shown to support *in vitro* phosphorylation and ubiquitination of katanin p60. Interestingly, silencing of EDD or Dyrk2 in HeLa cells caused G2/M arrest that was alleviated by concomitant silencing of katanin p60. Overexpressing katanin p60 had a similar effect that was overcome by co-expressing wild-type, but not kinase-inactive Dyrk2.

#### DNA damage responses

Mammalian cells have at least five uracil DNA glycosylases, among which UNG2 plays a critical role in repairing misincorporated or spontaneously generated uracil in genomic DNA [43]. Previously, the HIV protein Vpr was reported to load UNG2 onto VprBP for degradation (see below). More recently, however, Wen *et al.* found that UNG2 is directly targeted by CRL4^VprBP^ independently of Vpr, but Vpr can enhance VprBP-dependent UNG2 degradation [44]. Thus, silencing DDB1 or VprBP substantially increased UNG2 levels in transiently transfected 293T cells. Interestingly, when overexpressed, UNG2 causes DNA damage [45], colocalizes with the DNA damage marker γH2AX, and is toxic to cells [46], suggesting UNG2 is a potentially very important substrate of VprBP.

Mcm10 is a DNA replication initiation factor which recruits DNA polymerase α to the pre-RC to initiate DNA replication after CDK2 is activated [47]. UV radiation triggers Mcm10 proteolysis and causes stalling of DNA replication to facilitate DNA repair and avoid erroneous DNA synthesis [48]. Given the known role that CRL1^Skp2^ and CRL4^Cdt2^ E3 ligases play in irradiation-induced DNA damage responses, Kaur *et al*. suspected that one of these two E3 ligases might be responsible for Mcm10 degradation after UV-induced DNA damage [49]. Using a knock-down approach in HeLa cells, the authors excluded the involvement of CRL1^Skp2^, as well as Cdt2, in UV-induced Mcm10 degradation, but showed that silencing DDB1, Cul4, or Roc1 prevented the loss of Mcm10 after UV treatment. Systematic silencing of known CRL4 substrate recognition subunits identified VprBP as a likely candidate that links Mcm10 to the CRL4 ligase. Pull-down experiments confirmed Mcm10 association with the CRL4^VprBP^ complex, and delimited the interacting region to VprBP_864-1507_ (Figure 2). The CRL4^VprBP^ E3 ligase was also found to support Mcm10 ubiquitination *in vitro* and *in vivo.*

Hrecka *et al.* provided early evidence suggesting that silencing VprBP expression induced p53 target genes, such as p21, suggesting VprBP directly regulates p53-dependent transcription [50]. To test this possibility, Kim *et al.* analyzed transcription of p53-dependent genes induced after DNA damage, including p21 and Noxa, in MEFs in which VprBP expression was conditionally disrupted [30]. The authors showed that loss of VprBP expression increased p21 and Noxa expression levels induced by etoposide treatment. Using a cell-free assay to evaluate p53-dependent transcription from reconstituted nucleosomal substrates in reactions containing p300 HAT and Acetyl Co-A, the authors next showed that intact, but not denatured, VprBP suppressed p53-dependent transcription on nucleosomal, but not histone-free, DNA templates, as long as VprBP was added before p300, suggesting VprBP may block p300-dependent acetylation. This outcome was traced to VprBP’s ability to bind to unmodified histone H3 tails and was minimally mediated by VprBP_(751–1507),_ which encompasses the LisH and WD40 domains as well as the acidic C-terminus (Figure 2). Consistent with this finding, ChIP experiments demonstrated that knock-down of histone deacetylase (HDAC1) or VprBP, or etoposide treatment led to increased histone H3 acetylation and p53 occupancy at p53-dependent promoters; conversely, treatment with etoposide led to loss of HDAC1 and VprBP occupancy of the same promoters. These data suggested that VprBP physically associates with HDAC1 and p53. Evidence supporting this possibility was obtained using co-IP experiments, but the interacting regions in VprBP were not mapped. Because VprBP levels remained constant after DNA damage, the authors speculated that VprBP is subjected to post-translational modification after DNA damage to release VprBP bound to unmodified histone H3 tails. By screening a panel of kinases, acetyltransferases, and methyltransferases implicated in DNA damage responses, the authors identified DNA-PK as able to post-translationally modify VprBP. The phosphorylation site was mapped to Ser895, and an alanine substitution at this residue was demonstrated to attenuate p53-dependent transcription, and impair histone acetylation and release of VprBP from p53-dependent promoters in response to DNA damage. These data, taken together, led the authors to propose a model in which VprBP, in cooperation with HDAC1, removes and blocks acetylation of histone H3 tails to repress p53-dependent transcription. In response to DNA damage, DNA-PK phosphorylates VprBP at Ser895, which releases VprBP binding to histone H3, thereby allowing p53-dependent promoter activation.

The products of the recombination activating genes 1 and 2 (RAG1/2) are required to initiate V(D)J recombination, a form of site-specific DNA rearrangement that is responsible for assembling the immunoglobulin (Ig) and T cell receptor genes in B and T lymphocytes, respectively [51]. The N-terminal region of RAG1 (residues 1–383 of 1040), though not absolutely required for V(D)J recombination activity, is evolutionarily conserved and is necessary for efficient and high-fidelity rearrangement of the endogenous antigen receptor loci. The N-terminal region of RAG1 contains a RING domain that functions *in vitro* as an E3 ligase, but the *in vivo* functionality and physiological targets of RAG1 E3 ligase activity, if any, remains unclear. Moreover, since many RING-type E3 ligases form multi-subunit assemblies [52], RAG1 could plausibly associate with other accessory proteins to support its putative function as an E3 ligase. In support of this possibility, Kassmeier *et al.* showed that under mild purification conditions, novel proteins co-purified with full-length RAG1, one of which was identified as VprBP using mass spectrometry [53]. Subsequent experiments provided evidence that DDB1, Cul4A, and Roc1, but not EDD and Dyrk2, co-purify full-length RAG1, and that RAG1 association with the CRL4^VprBP^ complex is mediated primarily by RAG1 interactions with the WD40 domain of VprBP (Figure 2). This co-purified complex supported E3 ligase activity *in vitro*: this activity was not directed at the RAG proteins themselves, and was not obviously impaired by cysteine mutations in the RING domain of RAG1, leading the authors to speculate that RAG1 serves as a scaffold to recruit the CRL4^VprBP^ to ubiquitinate one or more as-yet unidentified substrates. Evidence for the physiological significance of VprBP function in lymphocytes was obtained by conditionally disrupting VprBP expression in the B cell lineage. These animals showed an arrest in B cell development at the pro-B-to-pre-B cell transition, which was associated with an increase in S phase and apoptotic cells. Evidence for abnormal repair of V(D)J recombination intermediates was also detected. Recently, we have found that B cell development can be partially rescued by enforced expression of Bcl2, but most mature B cells emerging in this case express the lambda light chain, rather than the more commonly expressed kappa light chain (Palmer and Swanson, unpublished data). This observation suggests that VprBP is not absolutely required for V(D)J recombination, although its involvement in the DNA repair phase of this process cannot yet be excluded. An intriguing alternative possibility is that VprBP regulates the accessibility or positioning of the kappa locus to permit RAG-mediated DNA rearrangements over the ~3 Mb span of the kappa locus. Because the lambda locus is only about 1/10^th^ the size of the kappa locus and contains many fewer gene segments, it may not require significant architectural changes to support V(D)J recombination, and therefore may undergo RAG-mediated rearrangement in a largely VprBP-independent manner.

#### Telomerase regulation

Telomerase is an enzyme responsible for adding DNA sequence repeats to chromosomal ends after DNA replication, which is necessary to maintain genomic stability and prevent gradual loss of genetic information with each cell division. The telomerase holoenzyme contains the *t*elomerase *r*everse *t*ranscriptase (TERT) subunit, telomerase RNA (TERC), and several accessory protein subunits [54]. Based on the observation that telomerase activity is not well correlated with TERT transcript levels among different cell types, and that TERT protein exhibits a short half-life in HeLa cells, Jung *et al*. speculated that TERT is regulated at the post-translational level by phosphorylation and/or ubiquitin-dependent degradation [55]. These authors identified Dyrk2 as a candidate TERT regulatory kinase by systematically overexpressing a panel of protein kinases and evaluating their ability to down-regulate TERT protein in HeLa cells. The authors subsequently showed that Dyrk2 interacts with and phosphorylates TERT at Ser457, and promotes its *in vitro* ubiquitination. Notably, however, kinase-inactive Dyrk2 binds but does not phosphorylate TERT, and does not trigger its degradation in cells. Consistent with these findings, depletion of Dyrk2 was shown to upregulate TERT expression and increased the half-life of the protein. Interestingly, although knock-down of Dyrk2 disrupted TERT association with VprBP and EDD, it had little effect on TERT-DDB1 interactions. This result is puzzling and remains unexplained. However, it raises the possibility that DDB1, rather than VprBP, is responsible for bringing TERT into proximity to EDD. In this regard, it is noteworthy that the region of VprBP interacting with TERT was not mapped, and the Dyrk2 and TERT truncation mutants used to map Dyrk2-TERT interactions were not evaluated for the presence of co-purifying DDB1 or VprBP, which could conceivably bridge Dyrk2 and TERT in the assay. Finally, the authors showed that TERT-Dyrk2 protein interactions were primarily limited to the G2/M phase, and were correlated with a decrease in TERT protein levels. In contrast, TERT-Dyrk2 association was not apparent during S phase, when telomerase activity is expected to be highest. Taken together, these data led the authors to suggest a model in which the TERT protein synthesizes a telomere repeat sequence at the early S phase of the cell cycle, and as the cell transitions to the G2/M phase, the EDD-Dyrk2 E3 ligase targets TERT protein for degradation to suppress telomerase activity.

#### Turnover of methylated proteins

*E*nhancer of *z*este *h*omolog 2 (EZH2) is a methyltransferase that specifically mediates histone H3K27 methylation and has been found to be deregulated in a variety of cancer types [56]. Given its likely role in tumorigenesis, Lee *et al.* were interested in identifying potential non-histone targets of EZH2 activity [25]. A computational screen of proteins carrying the amino acid sequence R-K-S in histone H3 that is targeted by EZH2 identified the orphan nuclear receptor RORα as carrying a potential acceptor site for methylation by EZH2. Subsequent studies demonstrated that EZH2 catalyzes monomethylation of RORα at K38 *in vitro*, and that loss of EZH2 activity or a K38A RORα mutation increased cellular RORα protein levels and extended its half-life, but did not alter RORα transcript levels, suggesting RORα methylation triggered its degradation. This hypothesis was further supported by the observation that wild-type, but not K38A, RORα protein ubiquitination products were increased in cells treated with the proteasome inhibitor MG132. To determine the putative ubiquitin ligase responsible for mediating RORα degradation, FLAG-RORα was purified from stably transfected HEK293 cells treated with MG132, and RORα−associating proteins were identified by mass spectrometry, yielding VprBP and DDB1 as RORα-interacting proteins. Subsequent studies established that Cul4 associated with RORα in a VprBP, DDB1, and EZH2-dependent manner, and showed that the VprBP-RORα interaction required active EZH2, suggesting the interaction was methylation dependent. The VprBP interacting region was mapped to a putative chromo-like domain in the N-terminal region (residues 562–593, Figure 2). The authors established the functional significance of RORα methylation-induced, ubiquitin-dependent degradation, by showing that transcription from RORα-dependent promoters and target genes was enhanced by a K38A RORα mutation or by silencing VprBP or EZH2 expression. Consistent with data suggesting EZH2 regulates RORα levels, the authors showed that breast cancer cells and tumors which overexpress EZH2 have lower RORα levels compared to normal controls. In addition, tumor cell growth in soft agar could be suppressed in the breast cancer cell line MCF7 by ectopically expressing RORα, inhibiting EZH2 activity, or silencing VprBP.

#### Neighboring cell competition

In the *Drosophila* model system, faster growing cells were discovered to induce apoptosis of surrounding slower growing cells in mixed cell culture by a process called “cell competition”. This process was found to depend on a tumor suppressor gene called *L*ethal *g*iant *l*arvae (Lgl) [57]. Using the mammalian Lgl homologue (mLgl2) as bait to search for factors that mediate Lgl-dependent cell competition, Tamori *et al.* identified VprBP as a Lgl-binding protein by mass spectrometry and showed that a C-terminal VprBP fragment containing the WD40 domains and the acidic region is minimally required to associate with mLgl2 [58] (Figure 2). In *Drosophila*, disruption of the VprBP homolog (called Mahjong) caused growth retardation and lethality at the late pupal stage. In both *Drosophila* and mammalian cells, loss of Mahjong/VprBP expression rendered cells susceptible to apoptosis initiated by neighboring wild-type cells, possibly through the activation of the JNK pathway. In this study, the authors did not report whether components of the CRL4 or EDD-Dyrk2 E3 ubiquitin ligases associate with the VprBP-mLgl2 complex, nor whether Lgl (or mLgl2) undergoes VprBP-dependent ubiquitination. Thus, the mechanism by which VprBP regulates Lgl to influence cell competition remains unclear.

### VprBP as a target of viral proteins

VprBP is targeted by the Vpr and Vpx accessory factors of primate lentiviruses, where they are thought to highjack the function of the CRL4^VprBP^ E3 ligase. The background and significance of these discoveries have been recently reviewed [2], and so will not be covered in depth here. What is notable for this review is that engagement of CRL4^VprBP^ by Vpr (but not Vpx) causes cell cycle arrest at the G2 phase. By contrast, Vpx association with the CRL4^VprBP^ E3 ligase is required to overcome host restriction and enable viral infectivity of myeloid cells. Vpr and Vpx appear to facilitate recruitment of different substrates to the CRL4^VprBP^ ligase: Vpr promotes loading of the uracil DNA glycosylases UNG2 and SMUG1 (*s*ingle-strand-selective *m*onofunctional *u*racil-DNA *g*lycosylase 1) [44,59,60], whereas Vpx recruits SAMHD1 (*s*terile *a*lpha *m*otif and *HD* domain-containing protein-1), a deoxyribonucleoside triphosphate (dNTP) triphosphohydrolase thought to inhibit HIV/SIV infection by maintaining cellular dNTP levels below the threshold required for robust viral reverse transcriptase activity [61-66]. As discussed above, Vpr may enhance the normal turnover of UNG2 by CRL4^VprBP^, whereas Vpx is thought to recruit SAMHD1 as a novel target of CRL4^VprBP^ to inhibit SAMHD1 enzymatic activity [67] and promote its degradation [61]. Notably, the Vpr-mediated G2 arrest phenotype does not depend on Vpr binding to UNG2 [68].

Interestingly, Wang *et al.* recently reported that Vpr also enhances TERT degradation mediated by VprBP [69], which may provide a mechanism to explain the downregulation of telomerase activity following HIV-1 infection [70,71]. These authors showed that knock-down of VprBP, but not Cul4A, alleviated Vpr-induced TERT degradation; a Vpr Q65R mutation that abolishes binding to VprBP also prevented Vpr-induced TERT degradation. The involvement of EDD-Dyrk2 E3 ligase was implied by the finding that EDD and DDB1 associate with Vpr in co-IP experiments, but their requirement for Vpr-induced TERT degradation was not formally established. While Dyrk2 was found to promote TERT ubiquitylation *in vitro*, its presence in Vpr immunoprecipitates and its requirement for Vpr-mediated TERT degradation in cells was not tested.

Based on an alignment of multiple VprBP sequences obtained from a yeast two-hybrid screen for Vpr-interacting proteins, Le Rouzic *et al.* concluded that Vpr interacts with the WD40 domain of VprBP [72]. This finding contrasts somewhat with data published by Zhang *et al.* showing that a VprBP fragment encompassing residues 636–1507, but not 1100–1507 (which contains most of the WD40 domain), co-IP’s Vpr from insect cells expressing both proteins [11].

Another viral protein that targets VprBP is human cytomegalovirus (HCMV) UL35 protein [28]. Like Merlin, UL35 binds to the far C-terminal acidic region of VprBP (Figure 2), and, like Vpr [73,74], UL35 overexpression triggers G2 arrest associated with cdc2 phosphorylation [28]. Silencing VprBP expression was shown to block UL35-mediated cdc2 phosphorylation, but its effect on cell cycle was not reported [28]. Although DDB1 was found to co-IP with UL35, interactions with other components of the CRL4 or EDD-Dyrk2 E3 ligases were not formally established. Thus, whether UL35 functions to inhibit or hijack one or the other E3 ligase currently remains unclear.

### Remaining questions

A number of very interesting questions regarding the normal function of VprBP and its targeting by viral accessory proteins remain to be answered. One important question centers on whether VprBP levels are limiting in the cell in comparison to the CRL4 and EDD-Dyrk2 E3 ligases, and if so, what conditions are necessary to enable VprBP to service these two E3 ligases if both are expressed in the same cell. Since VprBP is a demonstrated substrate of DNA-PK [30], one might imagine that phosphorylation, or an alternative post-translational modification, could provide a mechanism to influence which E3 ligase VprBP associates with at a given time within the cell. Of course, similar mechanisms may act on the core E3 ligase components themselves to prevent VprBP association, but this would presumably affect interactions with other substrate recognition subunits as well, and may therefore be less likely to occur. The dual specificity of VprBP for both the CRL4 and EDD-Dyrk2 E3 ligases also raises some concerns about whether effects observed in VprBP knock-down experiments can be uniquely attributed to inhibiting one or the other E3 ligase, particularly in situations where the CRL4 and EDD/UBR E3 ligases may have overlapping regulatory roles, such as during DNA damage responses [75-77].

Another central unanswered question pertains to the molecular mechanism by which Merlin binding to VprBP inhibits CRL4^VprBP^ activity. Among possible scenarios that could be imagined include competitive inhibition of substrate binding or blocking of conformational changes that facilitate ubiquitin transfer. In this respect, it is intriguing that Merlin binds to the same site on VprBP as UL35, but for reasons that remain unclear, does not trigger G2 arrest like UL35 [28]. The observation that Vpr also induces G2 arrest, but binds VprBP in a different location than UL35 (Figure 2), raises the possibility that Vpr and UL35 share a common mechanism for inducing G2 arrest that results from inhibiting, rather than redirecting, E3 ligase activity.

A third major question is the relationship between VprBP oligomerization and VprBP function and its implication for experimental interpretation. For example, Kim *et al.* reported that VprBP-mediated histone H3 binding and suppression of p53 target gene transcription was supported by VprBP_751-1507_, which contains the LisH and WD40 domains, but not by VprBP_910-1507_, which lacks the LisH region, leading the authors to conclude that the LisH region is responsible for histone H3 binding [30]. However, since the LisH region has been shown to mediate VprBP oligomerization [26], an alternative interpretation is that enhanced activity of VprBP_751-1507_ relative to VprBP_910-1507_ is attributed to the ability of VprBP_751-1507_ to oligomerize when binding histone H3 on nucleosomal DNA substrates, which could enhance otherwise weak intrinsic protein-protein interactions by avidity.

Given that DDB1 as well as several putative substrate and other interacting proteins associate with VprBP through the WD40 domain (Figure 2), another key question is the following: how can the WD40 domain accommodate both DDB1 and one or more of these other interacting proteins in the same macromolecular complex? One explanation may be that DDB1 interacts with only a portion of the WD40 domain in VprBP (which permits other proteins to access this region), and may also rely on contacts outside of the WD40 domain to maintain stable association with VprBP. In support of these possibilities, Kassmeier *et al.* showed that a VprBP_1-1000_ fragment lacking the WD40 domain associates with DDB1 [53], and residual DDB1 binding was detected with a VprBP_1-750_ fragment by others [22]. Structural studies in several laboratories suggest many DCAFs interact with DDB1 through two different interfaces: one mediated by part of the WD40 domain which makes hydrophobic contacts with the BPA domain of DDB1, and the other mediated by an amino-terminal alpha-helical motif that is inserted into a cleft between the BPA and BPC domains [18-21]. Although a comparable alpha-helical motif was not identified in VprBP using multiple sequence alignment approaches [18,21], the sequence motif is quite short (13–20 residues) and not highly conserved, so its presence may yet remain unrecognized somewhere within the first 750–1000 residues of VprBP. Furthermore, the WD40 domain in DDB1-associated substrate recognition subunits may contact other ligands using the face opposite the one used to bind DDB1. For example, DDB2 uses one side of its WD40 beta-propeller to contact DDB1 and the other side to bind substrate DNA [18,20]. The WD40 domain in VprBP may similarly be used to associate with both DDB1 and substrate molecules.

Another interesting question is whether VprBP functions solely to promote polyubiquitination and degradation of targeted substrates. For example, Mcm10 was shown to undergo VprBP-dependent polyubiquitination and degradation in response to DNA damage [49]. However, VprBP silencing alone does not affect Mcm10 protein levels, suggesting VprBP does not normally control Mcm10 turnover. Interestingly, Mcm10 is mono- to di-ubiquitinated during the G1-to-S phase transition, which promotes its function during DNA replication in budding yeast [78], and Mcm10 silencing causes cell cycle arrest at the G2 phase by checkpoint activation in mammalian cells [79]. These data raise the possibility that VprBP positively regulates Mcm10 function by mediating mono to di-ubiquitination in mammalian cells.

In summary, VprBP has been implicated in regulating a wide variety of normal cellular processes and is targeted by host and viral accessory proteins to inhibit or subvert its affiliated E3 ligases. At present, our understanding of whether or how VprBP can switch its allegiance between the CRL4 and EDD-Dyrk2 E3 ligases, especially in pathways where both E3 ligases may play a regulatory role, remains limited. Clarifying the existence and nature of this interplay will greatly improve our understanding of how VprBP regulates cellular processes through the CRL4 and EDD-Dyrk2 E3 ligases, and allow us to recognize the full implications of their inhibition or hijacking by host and pathogen-associated factors.

## Competing interests

The authors declare that they have no competing interests.

## Authors’ contributions

TN, KM, and PCS all contributed in drafting and editing the manuscript, and all authors read and approved the final manuscript.
